# Comparison between conventional and compressed sensing cine cardiovascular magnetic resonance for feature tracking global circumferential strain assessment

**DOI:** 10.1186/s12968-021-00708-5

**Published:** 2021-02-22

**Authors:** Tomoyuki Kido, Kuniaki Hirai, Ryo Ogawa, Yuki Tanabe, Masashi Nakamura, Naoto Kawaguchi, Akira Kurata, Kouki Watanabe, Michaela Schmidt, Christoph Forman, Teruhito Mochizuki, Teruhito Kido

**Affiliations:** 1 Department of Radiology, Ehime University Graduate School of Medicine, Hitsukawa, Toon, Ehime 791-0295 Japan; 2 Department of Radiology, Uwajima City Hospital, Uwajima, Japan; 3 Department of Cardiology, Saiseikai Matsuyama Hospital, Matsuyama, Japan; 4 Siemens Healthcare GmbH, Erlangen, Germany; 5 Department of Radiology, Yoshino Hospital, Imabari, Japan; 6 Department of Radiology, I.M. Sechenov First Moscow State Medical University, Moscow, Russia

**Keywords:** Compressed sensing, Cardiovascular magnetic resonance, Feature tracking, Myocardial strain

## Abstract

**Background:**

Feature tracking (FT) has become an established tool for cardiovascular magnetic resonance (CMR)-based strain analysis. Recently, the compressed sensing (CS) technique has been applied to cine CMR, which has drastically reduced its acquisition time. However, the effects of CS imaging on FT strain analysis need to be carefully studied. This study aimed to investigate the use of CS cine CMR for FT strain analysis compared to conventional cine CMR.

**Methods:**

Sixty-five patients with different left ventricular (LV) pathologies underwent both retrospective conventional cine CMR and prospective CS cine CMR using a prototype sequence with the comparable temporal and spatial resolution at 3 T. Eight short-axis cine images covering the entire LV were obtained and used for LV volume assessment and FT strain analysis. Prospective CS cine CMR data over 1.5 heartbeats were acquired to capture the complete end-diastolic data between the first and second heartbeats. LV volume assessment and FT strain analysis were performed using a dedicated software (ci^42^; Circle Cardiovasacular Imaging, Calgary, Canada), and the global circumferential strain (GCS) and GCS rate were calculated from both cine CMR sequences.

**Results:**

There were no significant differences in the GCS (− 17.1% [− 11.7, − 19.5] vs. − 16.1% [− 11.9, − 19.3; p = 0.508) and GCS rate (− 0.8 [− 0.6, − 1.0] vs. − 0.8 [− 0.7, − 1.0]; p = 0.587) obtained using conventional and CS cine CMR. The GCS obtained using both methods showed excellent agreement (y = 0.99x − 0.24; r = 0.95; p < 0.001). The Bland–Altman analysis revealed that the mean difference in the GCS between the conventional and CS cine CMR was 0.1% with limits of agreement between -2.8% and 3.0%. No significant differences were found in all LV volume assessment between both types of cine CMR.

**Conclusion:**

CS cine CMR could be used for GCS assessment by CMR-FT as well as conventional cine CMR. This finding further enhances the clinical utility of high-speed CS cine CMR imaging.

## Background

Left ventricular (LV) wall motion assessment is important in the management of ischemic heart disease and cardiomyopathy [1]. Various imaging modalities, including echocardiography [2], scintigraphy [3], computed tomography (CT) and cardiovascular magnetic resonance (CMR) [4] can be used for this purpose. Especially, retrospective electrocardiogram (ECG)-gated balanced steady-state free-precession (bSSFP) cine CMR can be used to assess the LV wall with high resolution and reproducibility [5–8]. Nonetheless, myocardial strain analyses using several advanced CMR techniques, including tagging [9], displacement encoding with stimulated echoes [10], strain encoding [11], and tissue phase mapping [12] have been shown to be superior to wall motion assessments for detecting differences in myocardial deformation. The global circumferential strain (GCS) assessment has been reported to be an independent prognostic indicator of both asymptomatic patients and those with heart failure [13, 14]. However, although these techniques can provide reference standard measurements of myocardial strain, they require additional time-consuming sequences that are often clinically impractical.

Feature tracking (FT), an algorithm that was originally developed for echocardiographic images, was introduced as a technique for the analysis and quantification of myocardial strain based on bSSFP cine images [15, 16]. This technique can also be applied to conventional cine CMR [17] to retrospectively analyze myocardial strain without additional acquisition of strain specific image sequence.

Recently, the compressed sensing (CS) technique with incoherent sampling and iterative reconstruction was suggested as a solution to accelerate CMR acquisition time [18–20]. The CS approach can drastically reduce the cine CMR scan time and improve patient compliance. In addition, previous studies demonstrated the utility of CS cine CMR for LV volume evaluation [21–23]. However, the effects of CS cine CMR imaging for FT strain analysis remains to be carefully studied. This study investigated the effects and adequacy of using CS cine CMR images for FT strain analysis compared to conventional cine CMR.

## Methods

### Study population

The present study was reviewed and approved by the ethics review board of Saiseikai Matsuyama Hospital, Matsuyama, Japan. Written informed consent was obtained from all participants. A total of 74 consecutive patients scheduled for CMR for various cardiac conditions were enrolled. All patients underwent CMR scanning including both conventional cine CMR and CS cine CMR sequences. Patients with arrhythmia (n = 6) and severely impaired breath-hold capacity (n = 3) were excluded from this investigation to avoid potentially poor cine CMR image quality. As a result, 65 patients were included in the analysis, and their characteristics are shown in Table 1.

**Table 1:** Clinical characteristics of the study population

Number of patients	65
Age (years)	70.9 ± 9.2
Gender (female/male)	18/47
Height (cm)	160.8 ± 9.5
Weight (kg)	62.1 ± 13.2
BMI (kg/m^2^)	23.8 ± 3.4
Heart rate (bpm)	62 ± 10
Clinical diagnosis	
Coronary artery disease	44 (68%)
Cardiomyopathy	14 (22%)
Valve disease	3 (5%)
Other	4 (6%)

Data are presented as n (%) and mean ± standard deviation, unless stated otherwise

*BMI* body mass index

### Cine CMR data acquisition

All CMR examinations were performed at 3 T (MAGNETOM Skyra; Siemens Healthineers, Erlangen, Germany) with 18-channel body matrix coil and 32-channel spine matrix coil. After scout images were obtained to plan the cardiac axis views, eight short-axis cine CMR images covering the entire LV were obtained. The segmented bSSFP sequence was used for the retrospective ECG-gated conventional cine CMR scans. Immediately afterward, a prospective ECG-triggered CS cine CMR scanwas performed using a prototype sequence. The data acquisition of CS cine CMR is single-shot using incoherent sampling of k-space. This is realized with a random distribution of the readouts on the Cartesian grid in k-space. Image reconstruction was performed with a non-linear, iterative SENSE-type approach implementing spatio-temporal regularization using redundant Haar wavelets [24]. The corresponding cost function was solved with a fast iterative shrinkage-threshold algorithm (FISTA) type optimization consisting of a gradient descend step for the quadratic terms and the evaluation of the proximal operator. The proximal operator is weighted with the regularization parameter, which was set to 0.001 and 0.005 for spatial and temporal regularization, respectively. Details regarding data acquisition and reconstruction of CS cine CMR have been described elsewhere [23]. We acquired the prospective CS cine CMR data for 1.5 heartbeats, definitely including the end-diastolic phase between the first and second heartbeat [23]. Both the temporal and spatial resolution, as well as the slice orientation, were kept identical between the two cine CMR protocols. In both conventional and CS cine scans, actual one cine phase was composed by 41.1 ms data acquisition (3.16 ms TR × 13 segments). And the conventional cine image was interpolated to 25 cine phases by calculated phase using retrospective gating with 1 heartbeat. The calculate phase interpolation was not applied for CS cine image using prospective triggering with 1.5 heartbeat. Detailed imaging parameters are listed in Table 2.

**Table 2:** CMR sequence parameters

	Conventional cine	CS cine
Sequence type	2D cine TrueFISP	2D cine TrueFISP
ECG mode	Retrospective gating	Prospective triggering
Repetition time (ms)	3.16	3.16
Echo time (ms)	1.4	1.4
FOV (mm^2^)	350 × 350	350 × 350
Image matrix	208 × 166	208 × 166
Spatial resolution reconstructed (mm^2^)	1.7 × 1.7	1.7 × 1.7
Temporal resolution (ms) (repetition time × segments)	41.1	41.1
Slice thickness (mm)	6	6
Slice numbers	8	8
Flip angle (degrees)	50	50
Bandwidth (Hz/pixel)	1145	1145
Number of segments	13	13
Cardiac phases	25	19–31
Breath-holds (n)	4	1
Acceleration factor	3	12.8
Iterative reconstruction (n)	-	80

*CS* compressed sensing, *ECG* electrocardiogram, *FOV* field of view

### LV volume and strain assessment

**Fig. 1:**
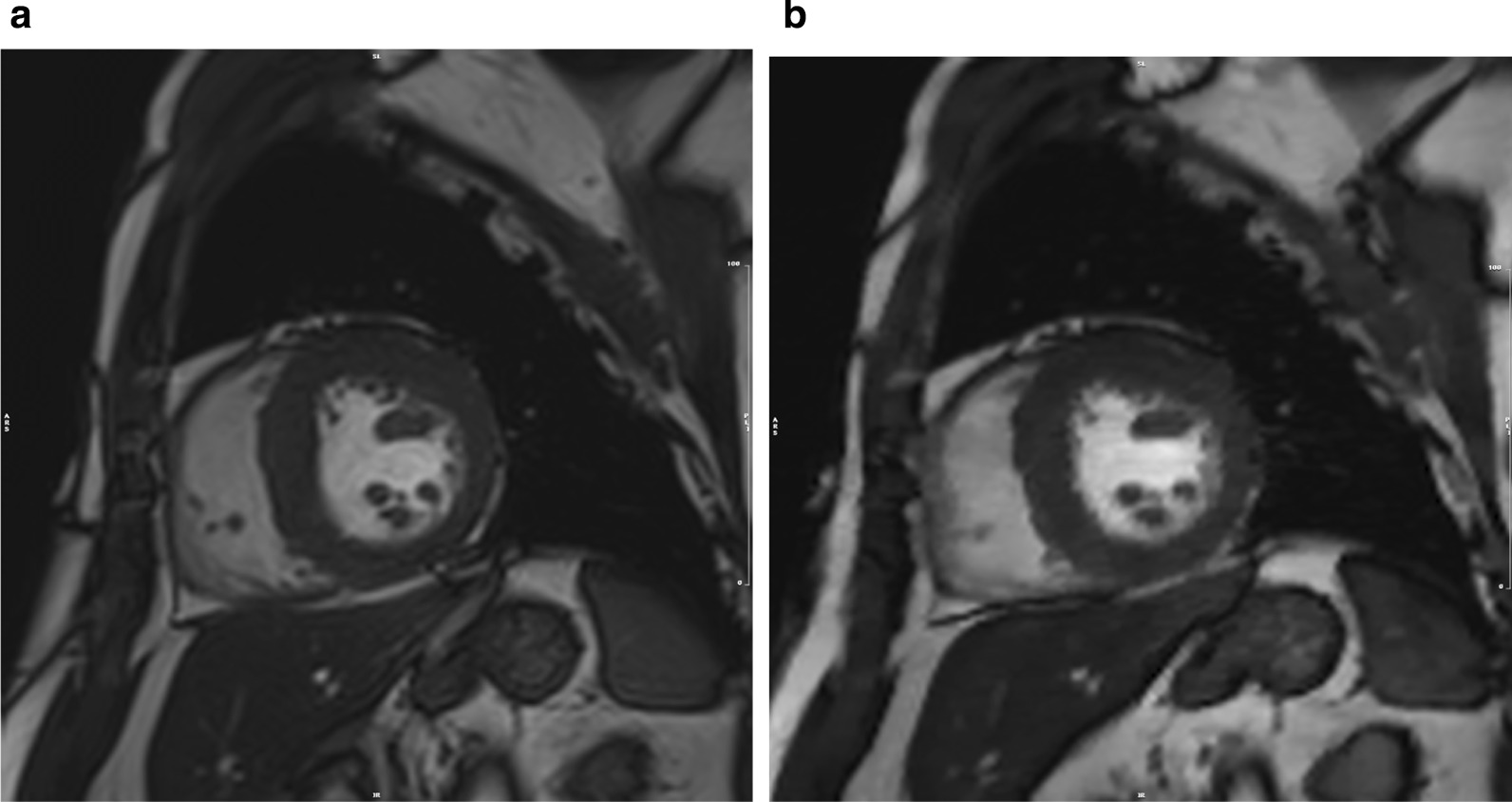
End-diastolic short-axis images obtained using conventional cine cardiovascular magnetic resonance (CMR) (**a**) and compressed sensing (CS) cine CMR (**b**)

Conventional and CS cine CMR images were analyzed using commercially available software (cvi^42^; Circle Cardiovascular Imaging, Calgary, Alberta, Canada) which has been previously validated [25]. LV volumes and mass were assessed by contouring the LV endocardial and epicardial borders in end-diastole and end-systole in both cine CMR images following the recommendations of Society for Cardiovascular Magnetic Resonance for post-processing [26]. The FT software algorithm could automatically draw both the endocardial and epicardial contours as well as track image features, including signal inhomogeneities, tissue patterns of the myocardium, and anatomic structures throughout the whole cardiac cycle. Additionally, the software allowed editing of the border when inadequate contours were reported (Fig. 1).

**Fig. 2:**
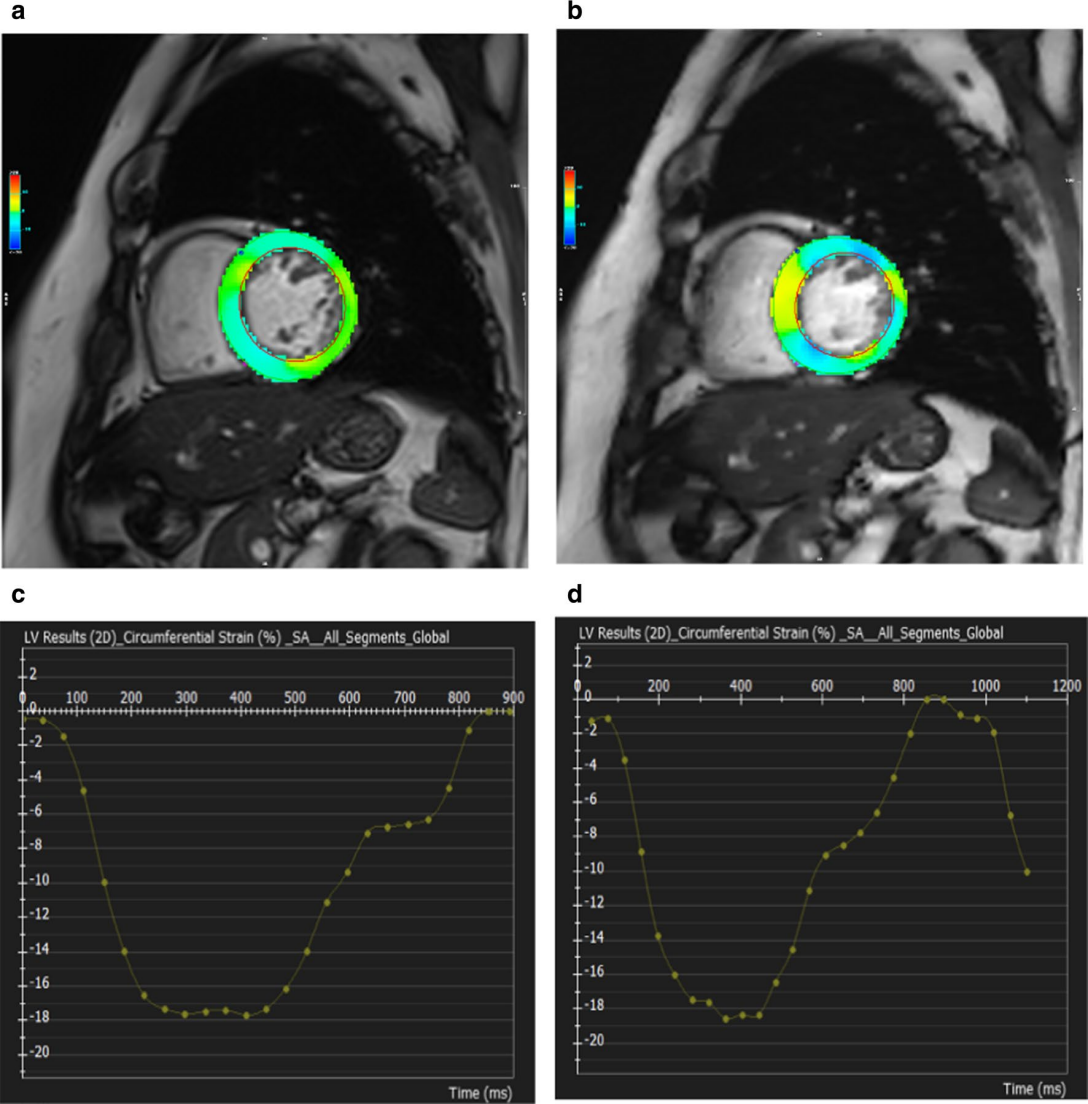
Example of strain assessment by CMR feature tracking (CMR-FT) in conventional (**a**, **c**) and CS cine CMR (**b**, **d**). The curves in the graph represent the similar global circumferential strains

To investigate the effects of CS imaging for FT strain assessment, the GCS and GCS rate were calculated using the two cine CMR images. Specifically, the GCS was calculated as the peak circumferential strain, using the mean of the eight-slice cine CMR images (Fig. 2). Given that the GCS is a measure of circumferential shortening from the baseline, it is typically a negative value. Therefore, greater circumferential shortening is indicated by an increasingly *negative* value.

The intra- and inter-observer variability of the FT strain assessment were performed in 20 randomly selected subjects. To measure the intra-observer variability, an interval of 4 weeks was chosen between the first and second analysis. Two independent blinded radiologists were employed to investigate the inter-observer variability.

### Image contrast assessment

To investigate the effects of using CS on image contrast, the contrast ratio (CR) between blood and myocardium was calculated in 20 randomly selected subjects. The regions of interest (minimum size: 80 mm^2^) were placed in both the midventricular septum and the LV lumen in an end-diastolic phase. The mean signal of the LV lumen was divided by that of the midventricular septum for CR measurement.

### Statistical analysis

Statistical analyses were performed using the commercially available software (JMP version 11; SAS Institute, Cary, North Carolina, USA). Continuous data are expressed as the mean ± standard deviation (SD) or the median (first quartile, third quartile) depending on the distribution. The Wilcoxon signed-rank test was used to compare the LV volume, the GCS and GCS rates obtained using the two cine CMR. The Spearman rank correlation coefficient and Bland–Altman analysis [27] were used to evaluate the correlation and agreement. Furthermore, intra-observer and inter-observer variability were evaluated by performing Bland–Altman analysis and calculating the intraclass correlation coefficient (ICC). The blood-myocardium CR determined by both cine CMR methods were compared using the paired t test. A p value < 0.05 was considered statistically significant.

## Results

All 65 patients successfully underwent scans using both conventional and CS cine CMR sequences. Furthermore, all conventional and CS cine CMR images were eligible for LV volume assessments and CMR-FT analyses. The total examination time was 113 ± 7 s and 24 ± 4 s for conventional and CS cine CMR, respectively (p < 0.001). For all LV volume measurements, no significant differences were found between conventional and CS cine CMR (Table 3).

**Table 3:** Left Ventricular volume measurements with Conventional and CS cine CMR

	Conventional cine (n = 65)	CS cine (n = 65)	Mean difference	P value
EDV (ml)	112.1 [95.7, 140.0]	111.4 [95.6, 154.9]	0.3 ± 8.3	0.84
ESV (ml)	47.2 [33.5, 78.6]	51.0 [34.3, 79.1]	-0.3 ± 6.4	0.36
SV (ml)	65.4 [52.8, 73.9]	62.1 [48.9, 76.4]	0.6 ± 9.8	0.70
Mass (g)	92.6 [75.8, 120.3]	92.2 [75.1, 117.3]	0.9 ± 5.9	0.29
EF (%)	58.6 [46.4, 66.3]	57.2 [46.2, 64.4]	1.1 ± 5.4	0.09

Data are presented as the medians [first quartile, third quartile]

Mean differences are presented as mean ± standard deviation

*EDV* end-diastolic volume, *EF* ejection fraction, *ESV* end-systolic volume, *SV* stroke volume

**Fig. 3:**
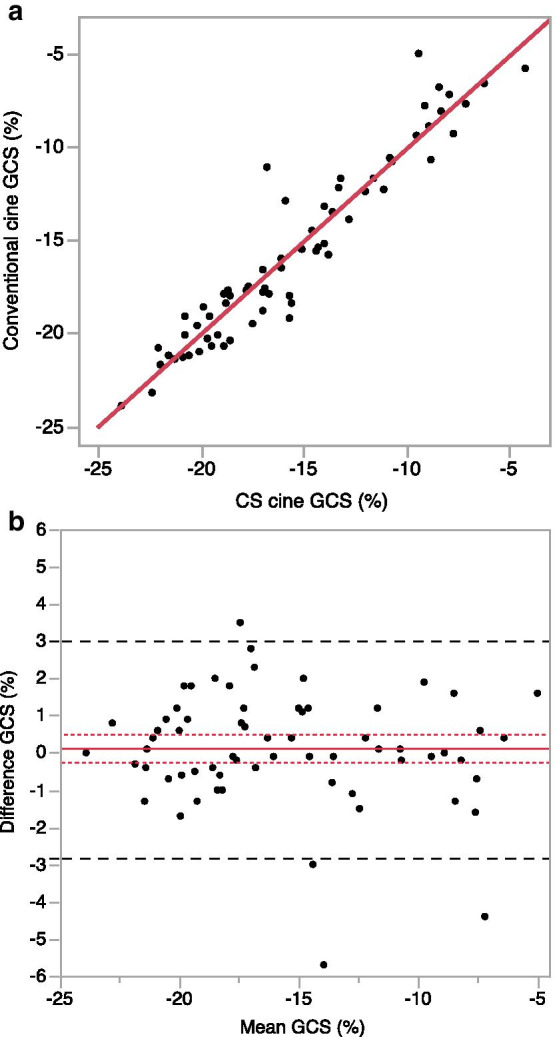
Scatter plots (**a**) and Bland–Altman plots (**b**) of global circumferential strain (GCS) measurements by CMR-FT. The solid line indicates the difference between the two sequences. The long-dashed lines represent the 95% agreement interval (i.e., the mean ± 1.96 SD). The short-dashed lines indicate the 95% confidence interval of the mean difference

There were no significant differences in GCS and GCS rate between conventional and CS cine CMR (Table 4). Furthermore, the GCS obtained using both methods showed good agreement according to the linear regression analysis (y = 0.99x − 0.24; r = 0.95; p < 0.001). Additionally, the Bland–Altman analysis revealed that the mean difference between conventional and CS cine CMR was 0.1% with limits of agreement between − 2.8% and 3.0% (Fig. 3).

**Table 4:** Results of GCS and GCS rate from conventional and CS cine CMR

	Conventional cine (n = 65)	CS cine (n = 65)	p
GCS (%)	− 16.1 [− 11.9, − 19.3]	− 17.1 [− 11.7, − 19.5]	0.508
GCS rate (s^−1^)	− 0.8 [− 0.6, − 1.0]	− 0.8 [− 0.7, − 1.0]	0.587

Data are presented as the median [first quartile, third quartile]

*CS* compressed sensing, *GCS* global circumferential strain

**Fig. 4:**
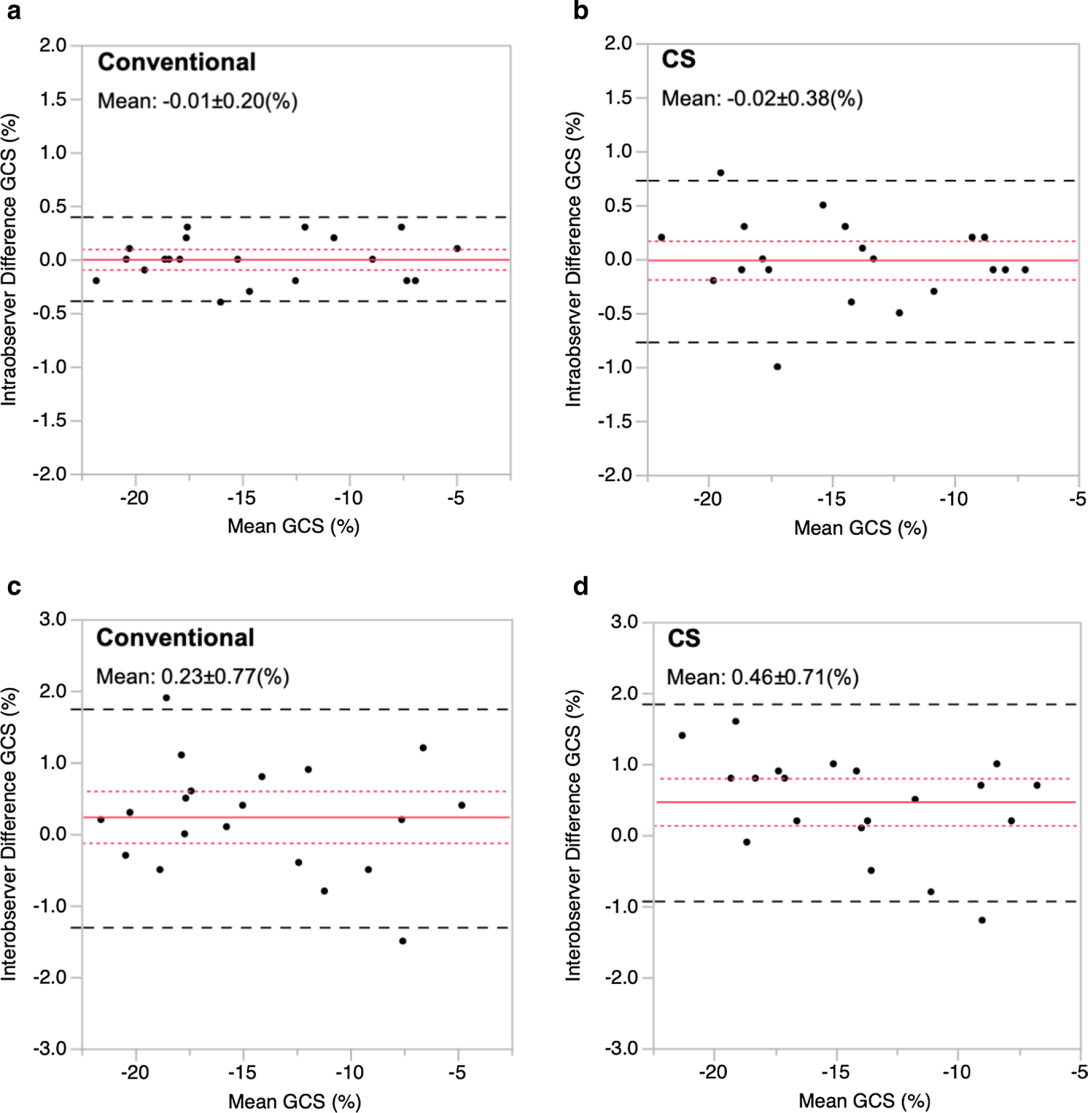
Bland–Altman plots of intra-observer variability of conventional (**a**) and CS (**b**) cine CMR. Bland–Altman plots of inter-observer variability of conventional (**c**) and CS (**d**) cine CMR. The solid line indicates the difference between the two sequences. The long-dashed lines represent the 95% agreement interval (i.e., the mean ± 1.96 SD). The short-dashed lines indicate the 95% confidence interval of the mean difference

Similarly, the intra- and inter-observer variability yielded a good agreement in both the conventional and CS cine CMR (Fig. 4), and their ICC were excellent (conventional cine CMR: ICC = 0.99 and 0.98 for intra- and inter-observer variability, respectively; CS cine CMR: ICC = 0.99 and 0.98 for intra- and inter-observer, respectively).

Finally, the blood-myocardium CR did not differ significantly between the two methods (conventional cine CMR: 5.0 ± 0.6; CS cine CMR: 4.9 ± 0.7; p = 0.956).

## Discussion

The strain assessment based on myocardial tagging is the most established technique utilized for measuring myocardial strain [13, 14]. However, the acquisition of additional images and the necessity of post-processing limits its use in routine clinical practice. Since its introduction [15], CMR-FT has been widely used for strain assessment in both clinical and research settings [16, 17, 28, 29]. CMR-FT can be applied to routine cine CMR; therefore, it has the potential to retrospectively evaluate myocardial strain in patients who did not undergo specific strain imaging.

The CS technique has been used for cardiovascular cine CMR. A previous study suggested using it to drastically shorten the imaging time while maintaining accurate LV volume quantification [23]. Because the widespread clinical use of CS cine CMR is desirable, it is essential to show that FT strain assessment results obtained using CS cine CMR are reproducible and comparable to those obtained using conventional cine CMR. The present study demonstrated that CS cine CMR could be used to assess GCS with high reproducibility as well as conventional cine CMR. This finding suggests that we can perform FT strain assessments using CS cine CMR and compare the results with those previously obtained using conventional cine CMR. The ability of CS cine CMR to assess both LV volume and strain within a short imaging time enhances its clinical utility.

Regarding the image features, the CS cine CMR images are associated with reduced noise but with a slightly pixelated and textured appearance compared with conventional cine CMR images due to its noise reduction process during the reconstruction step. Although the image features of CS cine CMR were slightly different from those of conventional cine CMR, good blood-myocardium CR was maintained and no significant differences were observed. Because that the FT algorithm is highly dependent on high contrast between the blood and myocardium [30, 31], similar results could be obtained during FT strain analysis.

Langton et al. evaluated FT strain based on images acquired with the CS real-time sequence that was prospectively triggered and acquired data for one heartbeat [32]. They showed there were highly consistent with conventional cine, but small systematic differences were observed. The prospective ECG-triggered cine CMR scan is useful, but capturing the complete end-diastolic data with this technique remains difficult because a finite time is required to detect the next ECG trigger [21, 22]. Because the strain value is indicated by the rate of change from the end-diastole (i.e., the reference point), the GCS may be underestimated using a one-heartbeat prospective cine CMR scan (i.e., the reference end-diastolic data may be missing). Therefore, it is crucial to acquire data of the whole cardiac cycle to perform accurate LV volume assessment and FT strain analysis using prospective cine CMR. In our study, prospective CS cine CMR data for 1.5 heartbeats were obtained to overcome this limitation and to capture the complete end-diastolic data between the first and second heartbeats.

We acquired CS cine CMR images using the comparable temporal and spatial resolution as conventional cine CMR in the current study; however, the effects of the differences in spatial resolution on FT have been reported [33]. Moreover, if the temporal resolution is too low, then it is expected that the strain value will be underestimated without capturing the complete diastole and systole phase. Furthermore, during CS cine CMR image reconstruction, the regularization setting also seems to affect the image quality and FT strain assessment results.

In our LV volume assessment, there was no significant difference, but CS cine tended to slightly overestimate end-systolic volume, probably as a result of the temporal regularization. Langton et al. found that good agreement to a conventional cine for GCS as in our study, but slightly less agreement for global radial strain^32^. Since radial strain reflects changes in myocardial wall thickness and is highly nonhomogeneous (increasing toward the endocardium), it is likely more sensitive to small differences in myocardial contouring. In our experience, too high regularization is likely to blur and smooth CS cine CMR images in spatial domain and to be insensitive with small motion in temporal domain. It would lead to an underestimation of strain. In this study, we used the fixed regularization value (0.001 for spatial regularization and 0.005 for temporal regularization), which was set based on the image quality of CS cine CMR, and this setting helped to maintaining the features necessary to calculate strain. We did not investigate the effects of changes in regularization during the FT strain analysis. Therefore, further detailed examinations of CS parameters are required and we should still be cautious standardized image acquisition to make FT strain measurements reliable and comparable.

### Limitations

There were some limitations to the present study. First, that the effects of using different types of CMR-FT analysis software were not assessed. Since the algorithm differs depending on the software, these inherent differences should be considered when comparing results obtained using different software [25]. Second, we did not obtain long-axis views using the CS cine CMR for global longitudinal strain (GLS) assessment, which may represent a strong predictor of outcomes and may potentially elucidate subtle changes in myocardial function [34, 35]. Further evaluations including GLS assessments using long-axis CS cine CMR are warranted to clarify the usefulness of CS cine CMR imaging for FT strain assessment.

## Conclusion

CS cine CMR images could be used for FT strain assessment with similar high reproducibility as conventional cine CMR. The ability of CS cine CMR to assess both LV volume and FT strain within a short imaging time enhances its clinical utility.

## Data Availability

Not applicable.

## Abbreviations

bSSFP: Balanced steady state free precession
CI: Confidence interval
CMR: Cardiovascular magnetic resonance
CR: Contrast ratio
CS: Compressed sensing
CT: Computed tomography
ECG: Electrocardiogram
EDV: End-diastolic volume
EF: Ejection fraction
ESV: End-systolic volume
FISTA: Fast iterative shrinkage-threshold algorithm
FT: Feature tracking
GCS: Global circumferential strain
GLS: Global longitudinal strain (GLS)
LV: Left ventricle/left ventricular
SD: Standard deviation
SV: Stroke volume
